# Underestimated Male Prevalence of Primary Biliary Cholangitis in China: Results of a 16-yr cohort study involving 769 patients

**DOI:** 10.1038/s41598-017-06807-7

**Published:** 2017-07-26

**Authors:** Xiaoli Fan, Tingting Wang, Yi Shen, Xiaotan Xi, Li Yang

**Affiliations:** Division of Gastroenterology & Hepatology, West China Hospital, Sichuan University, Chengdu, 610041 China

## Abstract

For primary biliary cholangitis (PBC), a sex ratio was reported to be significantly lower than previously cited in the West; we sought to evaluate sex ratio and long-term outcomes in PBC by studying a PBC cohort at a high-volume hospital from January 2001 to July 2016. A retrospective analysis including 769 PBC patients was conducted. The gender ratio was 6.1:1. Of the patients, 30.6% had one or more extrahepatic autoimmune (EHA) conditions. The proportion of patients with decompensated PBC at diagnosis increased from 25.0% in period 1 to 47.0% in period 4 (*p* < 0.05). Of the 420 patients without complications on presentation, the Kaplan-Meier estimate revealed distinct outcomes between non-cirrhotic PBC and cirrhotic PBC, with estimated mean survival times of 145.1 months and 104.5 months, respectively (*p* < 0.001). According to a subgroup analysis, gender and anti-mitochondrial antibody (AMA) status did not affect long-term prognosis, whereas patients with EHA conditions showed better prognoses. This study reveals evolving trends in male prevalence similar to their Western counterparts. Cirrhotic PBC patients were distinct from those with non-cirrhotic PBC at diagnosis based on difference in long-term outcome.

## Introduction

Primary biliary cholangitis (PBC) is a chronic autoimmune cholestatic liver disease characterized by the progressive destruction of small intrahepatic bile ducts. PBC often presents with cholestasis and the presence of anti-mitochondrial antibodies (AMAs) in the serum. The pathogenesis of PBC remains unknown; however, environmental, genetic and epigenetic factors are involved in the susceptibility of PBC^1, 2^. The global incidence and prevalence of PBC has been reported to range from 0.33 to 5.8 and from 1.91 to 40.20 per 100,000 people^3, 4^, and several studies have reported annual prevalence rates for several consecutive years that indicate increased rates in the West^4^. PBC was once a prime example of the characteristic sexual dimorphism in autoimmunity, and the female-to-male ratio was previously reported to be 10:1 in the West^1^. However, there is an evolving trend in the ratio of female-to-male incidence, with a value as low as 1.6:1 in the West^5, 6^.

With the decreasing incidence and prevalence of hepatitis B infection attributed primarily to vaccinations and antiviral treatments^7^, Chinese hepatologists have noted a recent increase in PBC^8^. The disease spectrum often differs between the East and West (e.g., with regard to hepatitis B, hepatitis C, and autoimmune hepatitis; AIH). Recently, several studies have explored the clinicopathological characteristics of patients with PBC in China, and two reports found that the female:male ratio was 11.6:1 and 10.9:1, respectively^9, 10^. However, these studies in China have been performed in developed areas, such as Shanghai and Beijing, rather than developing areas. Meanwhile, few investigations have conducted long-term follow-up assessments in large cohorts or examined the changes in PBC over time in China. The volume of inpatients at our institution is large; furthermore, because our patients originate from the western areas of China, our institution is an appropriate basis for a large population-based longitudinal study.

In 2015, the nomenclature for PBC was changed from “Primary Biliary Cirrhosis” to “Primary Biliary Cholangitis” to distinguish PBC from cirrhosis and to counter the misunderstandings, disadvantages and discrimination that might occur in patients’ daily lives due to that diagnosis^11^. This alteration also highlights doubts about whether patients with evident cirrhosis should be considered as having PBC.

Hence, in the present study, we performed a retrospective analysis to investigate the sex ratio and changes in Chinese PBC patients and evaluate the long-term outcomes of PBC patients for a 16-year cohort study.

## Results

### Gender, age and stage distribution at diagnosis across different periods

**Figure 1:**
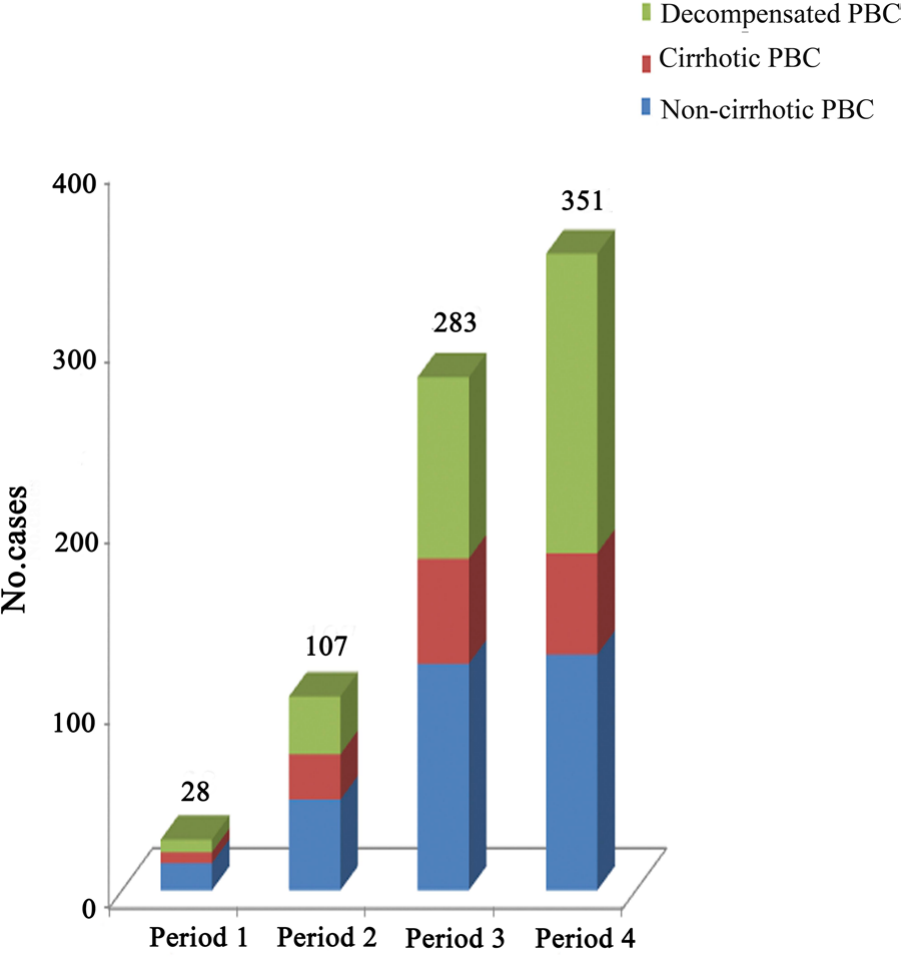
Time trends of PBC at different periods over the 16-year period from January 2001 to July 2016. The data were grouped over 4-year intervals.

A total of 769 cases of PBC from January 2001 to July 2016 were included in our analysis. Among these patients, 28 were diagnosed in period 1, 107 were diagnosed in period 2, 283 were diagnosed in period 3, and 351 were diagnosed in period 4 (Fig. 1). The gender, age and stage distributions of the cases across different periods are shown in Table 1. The mean age at diagnosis was 55.4 years (55.0 years for females and 58.4 years for males). No significant differences were observed with regard to age over the 4 periods (*p* = 0.068). The female-to-male ratio was 6.1, with 660 females and 109 males over 16 years (85.8% and 14.2%, respectively). The gender ratio remained relatively stable over the 16-year study (*p* = 0.576).

**Table 1:** Gender, age and stage distribution of PBC from January 2001 to July 2016.

	Period 1, 2001–2004 (n = 28)	Period 2, 2005–2008 (n = 107)	Period 3, 2009–2012 (n = 283)	Period 4, 2013-July 2016 (n = 351)	*P*
Sex					0.576
Male	6 (21.4%)	15 (14.0%)	43 (15.2%)	45 (12.8%)	
Female	22 (78.6%)	92 (86.0%)	240 (84.8%)	306 (87.2%)	
Age, years					0.068
<40	0 (0%)	14 (13.1%)	26 (9.2%)	19 (5.4%)	
40–60	15 (53.6%)	59 (55.1%)	160 (56.5%)	214 (61.0%)	
>60	13 (46.4%)	34 (31.2%)	97 (34.3%)	118 (33.6%)	
Age, years	57.3 ± 9.7	54.1 ± 12.1	55.5 ± 12.2	55.6 ± 11.6	0.552
Stages					
Non-cirrhotic PBC	15 (53.6%)	50 (46.7%)	125 (44.2%)	130 (37.0%)	0.086
Cirrhotic PBC	6 (21.4%)	25 (23.4%)	58 (20.5%)	56 (16.0%)	0.265
Decompensated PBC	7 (25.0%)	32 (29.9%)	100 (35.3%)	165 (47.0%)	0.001
Histological stages	4	9	15	60	0.276
I–II	1	3	9	36	
III–IV	3	6	6	24	

A decreasing trend was observed with regard to non-cirrhotic PBC from period 1 (53.6%) to period 4 (37.0%); however, this trend was not significant (*p* > 0.05). The proportion of decompensated PBC patients increased from 25.0% to 47.0% (*p* < 0.05).

### Distribution of clinical manifestations

The distribution of clinical manifestations was analyzed for all 769 patients over the 16-year cohort study. The most common manifestations were therapy requirements for complications (n = 301, 39.1%), followed by jaundice (n = 266, 34.6%), abnormal liver function without symptoms (n = 175, 22.8%), abdominal distension (n = 156, 20.3%), persistent fatigue (n = 122, 15.9%), pain in the right hypochondrium (n = 72, 9.4%), anorexia (n = 63, 8.2%), pruritus (n = 59, 7.7%), nausea (n = 49, 6.4%), and diarrhea (n = 30, 3.9%). Of the 301 patients with present or previous complications, 191 reported ascites, 177 reported variceal bleeding, 25 reported spontaneous bacterial peritonitis (SBP), 20 reported hepatic encephalopathy, 15 reported portal vein thrombosis, 7 reported septicemia or severe pulmonary infection and 3 reported hepatorenal syndrome.

### Distribution of extrahepatic autoimmune (EHA) diseases

Approximately 30.6% of all patients with PBC had one or more EHA condition. The most frequent EHA conditions observed were Sjögren’s syndrome (SS; n = 149, 19.4%); thyroid illnesses, including hypothyroidism, hyperthyroidism and thyroiditis (n = 28, 3.6%); systemic lupus erythematosus (SLE; n = 26, 3.4%); pulmonary interstitial fibrosis (n = 19, 2.5%); and rheumatoid arthritis (n = 19, 2.5%). In addition, autoimmune hematological system diseases, systemic sclerosis, polymyositis/dermatomyositis and others were observed in the EHA condition of PBC; however, these cases were sparse. A total of 42 patients were diagnosed with two or more EHA diseases (Table 2).

**Table 2:** Extrahepatic autoimmune diseases of PBC patients from January 2001 to July 2016.

	N (%)	Female (%)	Median age
Sjögren’s syndrome	149 (19.4%)	93.3	53.6
Autoimmune thyroid diseases	28 (3.6%)	85.7	55.7
Systemic lupus erythematosus	26 (3.4%)	96.1	45.2
Pulmonary interstitial fibrosis	19 (2.5%)	78.9	58.3
Rheumatoid arthritis	19 (2.5%)	94.7	54.3
Autoimmune hematological system diseases	12 (1.5%)	91.7	55.0
Systemic sclerosis	9 (1.2%)	88.9	51.6
Polymyositis/dermatomyositis	9 (1.2%)	100	59.2
Others	10 (1.3%)	100	55.6

### Histopathological assessment

A total of 709 (92.2%) patients tested AMA-positive in serum, whereas 60 (7.8%) tested AMA-negative. In total, 88 patients underwent liver biopsy for diagnosis or exclusion of other liver diseases. In total, 45 patients were stage I–II, and 43 were stage III–IV.

### Treatment for PBC

Ursodeoxycholic acid (UDCA; 13–15 mg/kg/d) prescriptions gradually increased from 60.7% in period 1 to 85.5% in period 4 (60.7%, 73.8%, 82.3% and 85.5%, for each period, respectively), showing an upward trend over the 16 years (*p* < 0.05). Corticosteroids and immunosuppressive agents were prescribed for a small number of patients with or without UDCA.

### Long-term survival and adverse outcomes

**Figure 2:**
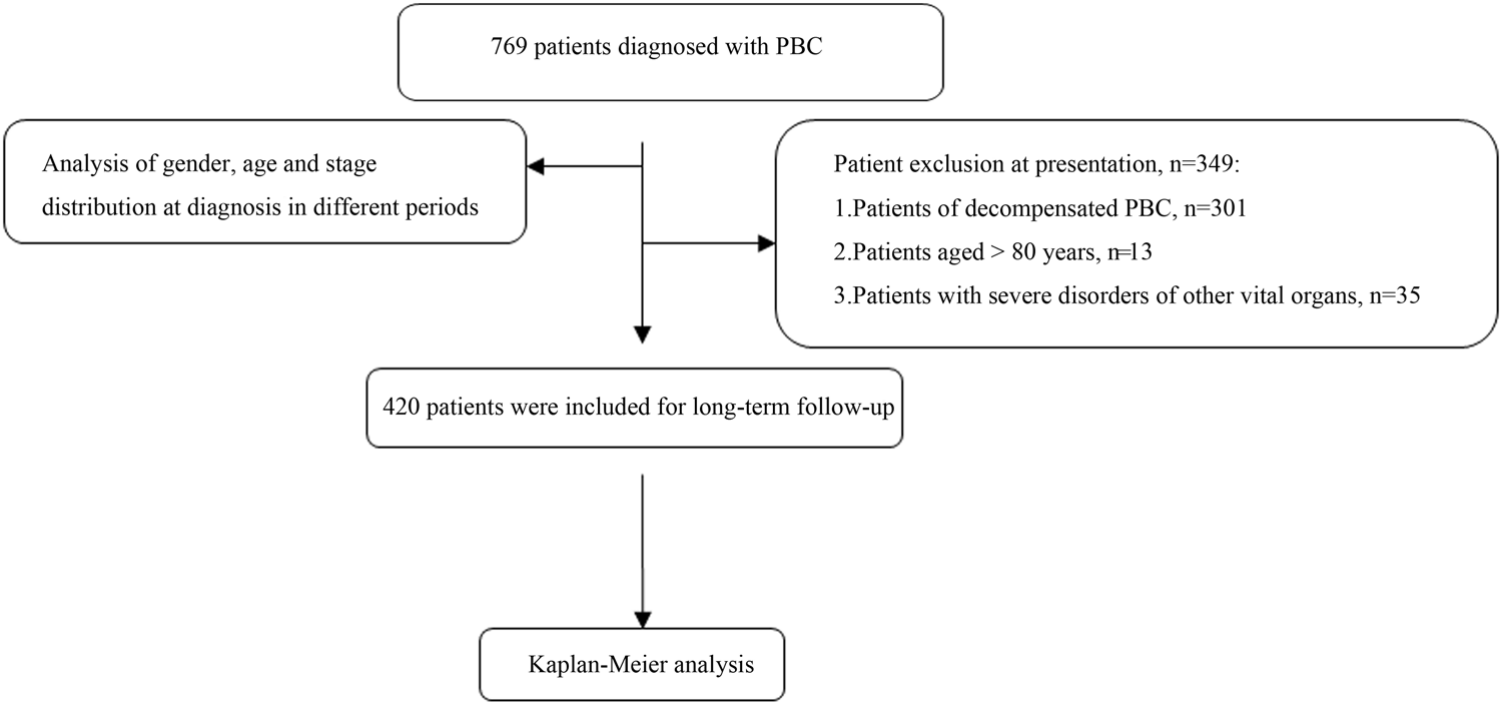
Patient selection flow chart.

Regarding the 420 patients without complications upon presentation at our medical center, 66 (15.7%) developed liver-related adverse events during the study period (up to September 1, 2016; Fig. 2). Of these patients, 29 (6.9%) experienced variceal bleeding at the end of the follow-up period or before death. An endoscopic intervention was performed for 8 patients, and TIPS was performed for 5 patients to treat variceal bleeding. Nevertheless, 4 cases died in the hospital, even after treatment with the combination of vasoactive agents and surgeries described above. Ascites (moderate to large) was diagnosed in 14 cases at the follow-up assessment, encephalopathy was diagnosed in 8 cases, and SBP was diagnosed in 4 cases. Liver transplantation (LT) was performed for 7 patients (6 women and 1 man) from 2001 to 2016, and the median duration was 36 months.

**Figure 3:**
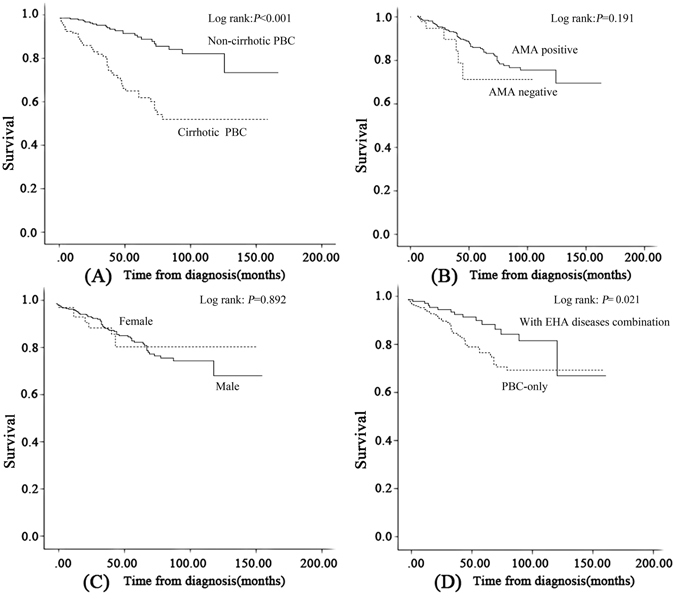
Kaplan–Meier plots for adverse outcome-free survival among patients with PBC stratified by stage, AMA status, gender and EHA disease combination. (**A**) PBC stage; (**B**) AMA status; (**C**) gender; and (**D**) EHA disease combination. A significant difference was observed between curves A and D (*p* < 0.05). Abbreviations: PBC, primary biliary cholangitis; AMA, anti-mitochondrial antibody; EHA conditions, extrahepatic autoimmune diseases.

Eleven patients died of adverse events due to cirrhosis or extrahepatic diseases. Of these deaths, 7 were cirrhosis-related; the causes of death were variceal bleeding (n = 4), encephalopathy (n = 2) and liver failure (n = 1). The extrahepatic diseases that caused patient deaths were cerebral infarction (n = 1), ovarian cancer (n = 1), pulmonary interstitial fibrosis (n = 1) and severe pulmonary infection (n = 1; Table 3). Importantly, the patients who died from pulmonary infection did not present with signs of cirrhosis at death. Figure 3 (panel A) shows the long-term prognosis of non-cirrhotic PBC and cirrhotic PBC on presentation, which had estimated mean survival times of 145.1 months and 104.5 months, respectively (95%CI: 136.9–153.3 months and 91.7–117.3 months, respectively). The Kaplan-Meier estimate showed that the outcomes were distinct between the two cohorts *(p* < 0.001). In addition, subgroup analysis of AMA status, gender and the combination of EHA conditions yielded *p*-values of 0.191, 0.892 and 0.021 based on log-rank tests, respectively (Fig. 3, panel B,C,D). These results demonstrated that cirrhotic PBC patients and those without EHA conditions had a significantly poorer prognosis.

**Table 3:** Newly occurring complications during follow-up in the PBC cohort (n = 420).

Outcomes	n (%)
Patients with adverse outcomes	66 (15.7%)
Variceal bleeding	29 (6.9%)
Ascites	14 (3.5%)
Encephalopathy	8 (1.9%)
Spontaneous bacterial peritonitis	4 (1.0%)
Liver transplantation	7 (1.7%)
Liver failure	3 (0.7%)
Liver-related death	7 (1.7%)
Death from causes unrelated to liver	4 (1.0%)

## Discussion

This single-institution series found a dramatic increase in the number of patients initially diagnosed with PBC over the last 16 years. This trend is inconsistent with other reports from several medical centers in China that have not reported increasing rates^8^. This increasing trend may be associated with a heightened awareness of PBC among physicians and the increased diagnostic rate in China.

A significant female preponderance is a widely accepted feature of PBC clinical description and the sex ratio corresponds to approximately 10:1. It has been revealed that haploinsufficiency for specific X-linked genes and involvement of X chromosome gene products participate in the female predisposition to PBC, as well as in other in autoimmune diseases^2, 12–14^. Hence, the findings of sex chromosome abnormalities facilitated the widely accepted striking female preponderance. Podda *et al*. for the first time proposed the underestimated male prevalence of PBC^15^, followed by 2 geoepidemiology researches in the West, where the female: male ratio could decrease to 1.6:1 from administrative databases in Sweden^5, 6^. The gender ratio in our study also corroborated the results of recent studies from South Korea and Japan (6.2:1 and 7.0:1, respectively)^16, 17^ and is much lower than those in previous reports from China^9, 10^. This study is the largest of Chinese PBC patients and implies that the evolving trend of female to male incidence in China is similar to that observed in Western populations^5, 6^. A potential bias cannot be completely excluded in the findings as case finding studies are based on clinical recognition and the initial clues leading to the suspicion of PBC. However, researchers have also found that Y chromosome loss associates with PBC in the male patient population recently^18^, which could support this finding. After all, the present analysis from China reflects a muted difference in the frequency of PBC between the sexes and places doubt on long-held beliefs about gender as a determinant of PBC.

Male and female PBC patients had similar mortality in the present study, in contrast to the findings of Lleo *et al*.^5^ and the Dutch PBC Study Group^19^. However, the observed differences in mortality have not been generally replicated, even in the West^20, 21^.

With respect to clinical characteristics, we found that PBC still predominantly affects middle-aged and elderly women, and the mean age of our patients at diagnosis was 55.4 years. This result corroborates previous reports on Caucasians and Asians^10, 16, 22^. Fatigue and pruritus are the most common symptoms among patients with PBC^1^. Although fatigue and pruritus at diagnosis were present in only 15.9% and 7.7% of our patients, respectively, these proportions were substantially lower than those in other reports from China^10, 23^, the United Kingdom^24^, and Canada^25^.

The proportion of decompensated PBC patients at diagnosis over the 4 time periods of our study increased with time, whereas the proportion of non-cirrhotic PBC patients seemed to decrease (difference was not significant). This trend might be related to increased awareness among primary physicians; Vast number of early patients do not seek help at our center. Hence, a bias exists, and our data do not represent the entire Chinese population. UDCA 13–15 mg/kg per day is recommended for all PBC patients, usually for life. An adequate prescription for UDCA might greatly change the clinical course of this disease^1, 26^. According to our retrospective study, UDCA availability increased throughout the 16-year study and reached 85.5% by period 4. However, corticosteroids and immunosuppressants were still prescribed for some treatment-naive patients in the present analysis, implying that the awareness of UDCA prescriptions in China should be enhanced.

Common EHA comorbidities include SS, autoimmune thyroid diseases, and SLE. The prevalence of these EHA conditions was high (30.6%), albeit lower than other reports regarding Caucasians, which reached as high as 61.2%^1, 27^; nevertheless, the present findings corroborated those from previous reports of Chinese patients^10^. The most common EHA comorbidity was SS (19.4%), followed by autoimmune thyroid diseases (3.6%) and SLE (3.4%). These percentages were lower than in previous studies, which have reported prevalences of 13.5–36.2%, 4.4–14.4%, and 1.5–3.7%^10, 27–29^, respectively. These differences might be attributable to case-finding methods and under-diagnosing as some reports from Asia found that the prevalence of EHA was much lower^16, 23^. Hence, systemic involvement and overlaps with other EHAs are lower but not infrequent in Chinese patients. Moreover, some special clinical and laboratory features (besides the common liver manifestations of PBC) might help to diagnose overlapping syndromes. A long-term prognosis revealed that having one or more EHA conditions influenced the incidence of liver-related adverse events; that is, survival was better for patients with EHA conditions than those with PBC alone. This finding is inconsistent with a report from Italy^27^. This discrepancy might be partly due to ethnicity differences; therefore, we deliberately considered this variable in our results. On the one hand, the use of corticosteroids or immunosuppressants to treat EHA conditions might alleviate autoimmune-reactions^1, 26, 30^. On the other hand, the co-existence of EHA conditions may relieve autoimmune inflammation and the destruction of small bile ducts. This latter effect might improve prognosis. This supposition is partially in line with Zhang *et al*., who found that the ALP, γ glutamyl transferase and immunoglobulin M values of patients with PBC and comorbid SLE were significantly lower than in the PBC-only group; however, these authors did not report patient prognosis^31^. The relationship between PSC and inflammatory bowel disease (IBD) may support our hypothesis^32, 33^.

The AMA profiles found in the present study were similar to those of previous reports. The survival rate revealed that AMA-negative patients had the same survival time as AMA-positive patients (*p* = 0.191 by a log-rank test), whereas Gunnar *et al*.^34^ found that AMA-negative patients had a significantly reduced survival free of liver-related complications, including transplantation and death, compared with AMA-positive patients. However, the majority of previous studies indicated no differences in prognosis^35, 36^.

At the end of the current study’s follow-up period, 66 (15.7%) patients developed liver-related adverse events. This finding is inconsistent with the findings of Shi *et al*.^10^. Variceal bleeding was the most common complication among our patients. Patients experienced this symptom first, implying that variceal status should be assessed at regular intervals, and primary or secondary prophylaxis should be conducted when necessary. LT was performed for only seven (1.7%) patients due to a limitation in medical resources. Considering the above, we suggest that the change in the definition of PBC from “primary biliary cirrhosis” to “primary biliary cholangitis” might be a milestone achievement or it may be arbitrary according to our data. Cirrhosis is the final stage of liver disease, and it often presents with end-stage complications and worse clinical outcomes. Treatments should be focused on UDCA responses in patients without cirrhosis; however, once a patient presents with the signs of liver cirrhosis or portal hypertension, clinicians must attempt to prevent and treat these complications, and delay the course to LT^37^. In the current study, the estimated mean survival times for patients with non-cirrhotic PBC and cirrhotic PBC were 145.1 months and 104.5 months, respectively. Thus, clinicians must determine the stage of PBC at diagnosis because whether a patient has cirrhosis might determine his or her long-term prognosis.

Although our study obtained promising results, certain limitations were also present. First, the study design of a single-center observation is subject to geographical restrictions that mitigate its representativeness and generalizability. Second, the risk factors associated with adverse outcomes still must be confirmed in another study. However, our results were based on the detailed clinical, laboratory and histology information of individual patients and not insurance claims or records; therefore, our rigorous inclusion criteria increased the robustness of our results.

Overall, our study found that the evolving trends of the female to male incidence of PBC in China are similar to those seen in Western countries. The prevalence of EHA diseases is lower among these patients than among those in the West. Our findings also provided important and clear information showing that the change in PBC from “cirrhosis” to “cholangitis” might obscure prognoses. It is important for clinicians to determine the stage of PBC at diagnosis. Moreover, we await accurate data on the rates of Chinese PBC patients.

## Methods

### Study population

This study was based on a retrospective review of all patients diagnosed with “primary biliary cirrhosis” or “primary biliary cholangitis” and followed up at West China Hospital, which is affiliated with Sichuan University, from January 1, 2001 to July 31, 2016. Medical profiles, including patient demographics and clinical features, were analyzed (ClinicalTrials.gov no.: NCT 02917408). A total of 769 consecutive patients with PBC were included in our retrospective analysis. To prevent repetitive cases from interfering with the data analysis, an incident case referred to a newly diagnosed case for which no previous records of PBC could be found at our center. PBC was diagnosed when at least two of the following three criteria were fulfilled: (1) serum alkaline phosphatase (ALP) levels at least 2 times the normal upper limit; (2) the presence of AMAs in serum; and (3) representative histological manifestations of portal area inflammation and bile duct injury^38^. Patients with AIH, viral hepatitis or other causes of liver damage were excluded. All parameters were examined at the Department of Laboratory Medicine of West China Hospital, which is certified by the College of American Pathologists (CAP). Changing trends for newly diagnosed patients with PBC were analyzed across 4 consecutive time periods: from 2001 to 2004 (period 1), 2005 to 2008 (period 2), 2009 to 2012 (period 3), and 2013 to July 2016 (period 4). The study was approved by the Ethics Committee of the West China Hospital. Methods were performed in accordance with the approved guidelines. All subjects provided written informed consent before enrolment.

### Baseline characteristics, follow-up and end point

Two researchers (Xiaoli Fan and Tingting Wang) carefully recorded the demographic parameters, clinical characteristics (age, sex, clinical signs, manifestations, comorbidities and imaging findings), and treatments. Cases were classified into 3 types: non-cirrhotic PBC (i.e., patients without signs of cirrhosis or portal hypertension on imaging or with a stage I–II histological assessment); cirrhotic PBC (i.e., patients with signs of cirrhosis or portal hypertension or with a stage III–IV histological assessment); or decompensated PBC. The third group was characterized by the presence of dramatic and life-threatening complications, such as ascites, variceal hemorrhage, severe infection, hepatic encephalopathy, hepatopulmonary syndrome or hepatorenal syndrome.

Long-term, adverse-outcome-free survival was evaluated via a direct phone call, an assessment of the medical records in our database, or both. An adverse outcome was defined as the occurrence of one or more of the following: complications of liver cirrhosis, liver failure, and LT. The exclusion criteria for the follow-up cohort included patients older than 80 years of age, those with severe disorders of other vital organs and those with decompensation events at diagnosis at our center. A total of 420 patients were enrolled in our final cohort for survival analysis.

### Histological assessment

A liver biopsy assessment was necessary for diagnosis when the AMAs in the serum were absent. The histological features of PBC included florid biliary damage, ductopenia, lymphocyte aggregation in the portal areas, granulomas and cholestasis. The histological assessment was evaluated according to Ludwig’s classifications: Stage I = portal inflammation, stage II = extension to the periportal areas, stage III = septal fibrosis or inflammatory bridging, and stage IV = cirrhosis^39^.

### Statistical analysis

The descriptive statistics are presented as the mean ± standard deviation (SD) or median (interquartile range). Categorical variables are reported as counts and percentages. The differences regarding continuous variables among groups were compared with a one-way analysis of variance. Categorical variables were compared using χ2-tests. All of the analyses in the current study were 2-tailed, and a *p*-value of <0.05 was considered significant. A Kaplan-Meier analysis was used to assess survival. All analyses were computed using SPSS (SPSS version 22.0 for Windows, IBM Corp., Armonk, NY, USA).
